# Defining traumatic brain injury in children and youth using International Classification of Diseases version 10 codes: a systematic review protocol

**DOI:** 10.1186/2046-4053-2-102

**Published:** 2013-11-13

**Authors:** Vincy Chan, Pravheen Thurairajah, Angela Colantonio

**Affiliations:** 1 Toronto Rehabilitation Institute, University Health Network, 550 University Avenue, Toronto M5G 2A2, ON, Canada; 2 University of Toronto, 160-500 University Avenue, Toronto M5G 1V7, ON, Canada

**Keywords:** Children and youth, Definition, ICD-10, Prevention, Protocol, Systematic review, Traumatic brain injury

## Abstract

**Background:**

Although healthcare administrative data are commonly used for traumatic brain injury research, there is currently no consensus or consistency on using the International Classification of Diseases version 10 codes to define traumatic brain injury among children and youth. This protocol is for a systematic review of the literature to explore the range of International Classification of Diseases version 10 codes that are used to define traumatic brain injury in this population.

**Methods/design:**

The databases MEDLINE, MEDLINE In-Process, Embase, PsychINFO, CINAHL, SPORTDiscus, and Cochrane Database of Systematic Reviews will be systematically searched. Grey literature will be searched using Grey Matters and Google. Reference lists of included articles will also be searched. Articles will be screened using predefined inclusion and exclusion criteria and all full-text articles that meet the predefined inclusion criteria will be included for analysis. The study selection process and reasons for exclusion at the full-text level will be presented using a PRISMA study flow diagram. Information on the data source of included studies, year and location of study, age of study population, range of incidence, and study purpose will be abstracted into a separate table and synthesized for analysis. All International Classification of Diseases version 10 codes will be listed in tables and the codes that are used to define concussion, acquired traumatic brain injury, head injury, or head trauma will be identified.

**Discussion:**

The identification of the optimal International Classification of Diseases version 10 codes to define this population in administrative data is crucial, as it has implications for policy, resource allocation, planning of healthcare services, and prevention strategies. It also allows for comparisons across countries and studies. This protocol is for a review that identifies the range and most common diagnoses used to conduct surveillance for traumatic brain injury in children and youth. This is an important first step in reaching an appropriate definition using International Classification of Diseases version 10 codes and can inform future work on reaching consensus on the codes to define traumatic brain injury for this vulnerable population.

## Background

Traumatic brain injury (TBI) is defined as ‘an alteration to brain function, or other evidence of brain pathology, caused by an external force’ [1]. It is a leading cause of death and disability worldwide and the physical, cognitive, and psychosocial impact and long-term effects of TBI have been well documented [2,3]. It is estimated that by the year 2020, TBI will exceed many diseases as the major cause of death and disability [4]. Recent data from the Centers for Disease Prevention and Control (CDC) in the United States showed that the highest rates of TBI-related emergency department (ED) visits from 2002 to 2006 were among children aged 0 to 4 years and older adolescents aged 15 to 19 years. Approximately half a million ED visits for TBI were made annually by individuals 14 years and under [5]. Colantonio and colleagues also showed that between fiscal years 2003/04 and 2009/10 in the province of Ontario in Canada, 36% of all TBI-related ED visits and 16% of all TBI-related acute care admissions were among children and youth 18 years and under [6].

The International Classification of Diseases (ICD) [7] is used to identify cases in healthcare administrative data. The ICD is the ‘standard diagnostic tool for epidemiology, health management, and clinical purposes’. Currently, the ICD is in its tenth version (ICD-10) and came into use by World Health Organization Member States in 1994. The majority of Canada’s provinces and territories began using ICD-10 in the year 2001 and by the year 2006, it was completely implemented [8]. It is also currently being used in other countries, including Australia, France, United Kingdom, and Germany [9]. In the United States, the new ICD-10 compliance date is set to October 1, 2014 [10]. The ICD-10 replaced the ninth version (ICD-9), which was very different in coding structure and concept. Specifically, the ICD-9 codes lacked detailed information on conditions and did not provide the option to distinguish between first and subsequent occurrences of the same condition [10,11]. Moreover, the ICD-10 provides clinical information, greater specificity, and updated medical terminology and classification of diseases since the creation of ICD-9 in 1979 [10-13]. For example, the ICD-10 devotes an entire spectrum of codes (S00 to S09) from Chapter XIX, Injury, Poisoning, and Certain Other Consequences of External Causes, to head injury while head or brain injury ICD-9 codes were interspersed within the Injury and Poisoning 800 to 999 codes. In addition, more specific codes related to head injury were created in ICD-10. It was not possible to distinguish superficial injury of the scalp from superficial injury of the face and neck in ICD-9; however, the ICD-10 provides the option to specify superficial injury to the scalp as S00.0. It is also important to note that certain codes were lost in the ICD-10, such as the code for shaken baby syndrome. While there was a specific ICD-9 code for shaken baby syndrome (995.55), the ICD-10 equivalent is an ICD-10 S06 code with Y07, which is a maltreatment syndrome code. As such, a diagnosis of shaken baby syndrome would be missed if a S06 code were not coded with Y07 [14].

Although healthcare administrative data are often used for TBI research, there is currently no consensus on the ICD-10 codes to define TBI. As a result, reported incidence of TBI varies greatly between countries [15]. A recent opinion piece published in *Nature Reviews Neurology* by Roozenbeek and colleagues suggested that reported incidence in the literature is likely an underestimate, owing to the limitations of ICD codes, such as variability and sensitivity of the coding and the fact that ICD codes were intended for administrative purposes rather than epidemiological research [16]. Research on the ICD-9 showed significantly lower rates among young adults, males, and those with less severe TBI using the ICD-9 CDC TBI surveillance codes by comparison with medical record data [17]. This finding was more recently demonstrated by Carroll and colleagues in 2012 [18]; in addition, they found that 20% of their patients were assigned ICD-9 codes that did not describe the specific type of TBI. Deb also found that ICD-10 codes did not detect all head injury admissions. Records from the accident and emergency department’s case register in the UK were compared with a list collected from the health authority’s central database using the ICD-10 codes. Deb found that only 37% of records from the case register appeared in the ICD-10 list while 41% of records in the ICD-10 list appeared in the case register [19]. Finally, St. Germaine-Smith and colleagues conducted a systematic review on the optimal ICD codes to study neurological conditions and found that inclusion of less specific TBI codes resulted in a lower positive predictive value; they noted a study that found low sensitivity and positive predictive value [20]. However, this study was not restricted to ICD-10 codes and concluded that the ‘ICD codes used and the diagnostic accuracy were too varied to allow recommendations regarding the best case definition’. This is of particular concern for efforts to prevent TBI in these vulnerable populations and has implications for resource allocation.

This protocol is for a systematic review of the literature to explore the range of ICD-10 codes that are used to define TBI among children and youth aged 19 years and under. Children and youth are at a critical developmental stage of their lives, in which adverse events may result in serious negative long-term consequences. In the case of TBI, there are unique features of a pediatric patient [21,22], including vulnerability of the still-developing brain and skull, which makes this population more vulnerable to brain injury and negative long-term consequences [23]. These negative outcomes include psychiatric illnesses [24,25] and deficits in cognition, attention, and executive function [26-30]. Finally, the pediatric population may be at risk of trauma from abusive situations, including shaken baby syndrome, which may lead to a TBI [31] and cannot be self-reported, owing to the child’s limited communication abilities. Current systematic reviews have brought attention to the importance of accurate codes [20,32]; however, none specifically examined ICD-10 codes for the children and youth population. Surveillance of TBI in children and youth is crucial and the availability of accurate information is essential for evaluating, planning, and transforming healthcare systems to better address the needs of this population. As such, it is important to identify the range of ICD-10 codes that are used to identify children and youth in order to capture this population accurately and appropriately in healthcare administrative data for research purposes.

## Methods/design

### Search strategy

The following databases will be searched for relevant articles:

1. MEDLINE (1946 to present).

2. MEDLINE In-Process (present).

3. Embase (1980 to present).

4. PsychINFO (1805 to present).

5. CINAHL (1981 to present).

6. SPORTDiscus (1800 to present).

7. Cochrane Database of Systematic Reviews (2005 to present).

Additional file 1 provides the search strategy associated with each database. Searches will be limited to the year 1992 to present, as the focus of this article is on selecting ICD-10 codes and work on ICD-10 was completed in 1992. Grey literature will also be searched using ‘Grey Matters, A Practical Search Tool for Evidence-Based Medicine’ [33] and Google. Search terms will also be derived using relevant published reviews as guides [20,32,34-38]. This review will purposely include articles that examine head injury, as the terms ‘head injury’ and ‘brain injury’ are sometimes used interchangeably even though they describe different conditions. As such, including articles that examine head injury will decrease the chance that some relevant articles will be missed and can assist in elucidating the codes that are primarily used to identify head injury versus brain injury in this population. The literature will also be searched for evidence of a relationship between brain injury and specific conditions, to inform the appropriateness of including specific codes in the case definition of TBI in children and youth. Where evidence suggests a relationship between brain injury and the condition the code described, it will also be suggested for inclusion in the definition of TBI in this population.

### Study selection

For all databases, two reviewers will independently assess all title and abstracts for fulfillment of predetermined eligibility criteria. A first screen, the title and abstract screen, will be conducted on all retrieved articles. Those that pass the first screen must have a full-text version available and meet at least one of the following inclusion criteria:

1. Use ICD-10 codes to define concussion, acquired TBI, head injury, or head trauma among children and youth 19 years or under.

2. Use ICD-10 codes to define concussion, acquired TBI, head injury, or head trauma but do not define the age of the population.

3. Examine concussion, acquired TBI, head injury, or head trauma among children and youth aged 19 years and under but do not define the data source.

4. Examine concussion, acquired TBI, head injury, or head trauma but do not define the age of the population or data source.

Articles that meet any of these first screen inclusion criteria will be included for the second screen, which will be a full-text screen. Two reviewers will independently assess all full-text articles for fulfillment of predetermined eligibility criteria. Articles that will be included for the systematic review must use ICD-10 codes to define concussion, acquired TBI, head injury, or head trauma among children and youth aged 19 years and under. These ICD-10 codes must be listed in the article or provided as supplemental information available to download online. The articles must clearly state the definition of a concussion, TBI, head injury, or head trauma (for example, articles that provide one definition for TBI and head injury would be excluded, as these are different conditions). Also, studies that are not limited to children and youth aged 19 years and under must have data stratified by age groups, such that findings for individuals aged 19 years or under are clearly presented. Where age categories overlap with the adult population, over the age of 19, children and youth must comprise at least half of the age category in the age range. For example, an article that stratifies the data by age groups with 15 to 24 year olds as the youngest age group would be included because 15 to 19 years of age is 50% of the 15 to 24 years age range. Conversely, an article with the youngest age category of 18 to 25 years of age would be excluded because ages 18 and 19 years are less than 50% of the 18 to 25 years age range.

The reference lists of included full-text articles will also be hand-searched. An expert in the field of administrative data and TBI will be consulted, to ensure that no additional studies are missed with the use of the above search strategy. The study selection process along with reasons for exclusions at the full-text level will be presented using a PRISMA study flow diagram.

### Data extraction

Study data will be abstracted independently by two reviewers and include:

1. Author and publication year.

2. ICD-10 codes used to define concussion, acquired TBI, head injury, or head trauma.

3. Source of data.

4. Year of study.

5. Location of study.

6. Age of study population.

7. Range of incidence.

8. Purpose of study.

Additional file 2 provides template of tables in which results will be synthesized.

### Quality assessment

Quality assessment of the codes will be determined by whether the ICD-10 codes that are used to define TBI are validated. Findings will be categorized into ‘yes’ or ‘no’ (in reports, ‘no’ will be replaced by ‘unclear’, as codes may be validated in other studies but not stated in the reports). Validation of codes is critical, as it provides information on the accuracy of coding and agreement for diagnoses. Given the objective of this review and the importance of validated codes, this quality assessment of the codes was preferred over more standard quality assessment tools.

### Analyses

ICD-10 codes used to define concussion, acquired TBI, head injury, or head trauma will be abstracted and each article will be categorized by the type of TBI and head injury (mild TBI or concussion, TBI, severe TBI, head injury, and abusive head trauma), study purpose (to identify incidence and trends or to identify TBI-related deaths), and target population (categories will be created according to results obtained from articles that meet the inclusion criteria of this review).

A range of ICD-10 codes used to define TBI in children and youth will be identified. Codes that are used consistently among TBI articles and, in particular, articles that examine TBI in children and youth aged 19 years or under will be suggested for inclusion in the definition of TBI in this population.

## Discussion

To the best of our knowledge, this is the first protocol for a systematic review that explores the range of ICD-10 codes to define TBI specifically in children and youth. This focus on children and youth provides the opportunity to address coding issues that are unique to this population, which has the potential to be undercounted owing to reporting difficulties, especially among infants. This review provides a summary of the range of ICD codes used and implications in terms of estimates. Further, it provides evidence for discussion on how best to use ICD codes for different goals. It provides a baseline of research at a specific point in time, as we move forward to code in a more standard way internationally.

The search strategy for this systematic review intentionally includes a broad range of terms that may be related to TBI (for example, head injury, facial fractures), as testing of searches revealed that relevant articles would be excluded if we restricted search terms to ‘traumatic brain injury’ only. Moreover, terms relevant to retinal hemorrhage were intentionally included. Many studies have identified retinal hemorrhage as a predictor of inflicted TBI in infants and young children [39], which includes shaken baby syndrome [40-44] and abusive head trauma [45,46]. Shaken baby syndrome is a form of abusive head trauma and inflicted TBI [47], resulting in intracranial hemorrhage [48]. It has been stated that retinal hemorrhage is present in 50% to 100% of cases and often clinches a diagnosis of shaken baby syndrome [43]. Retinal hemorrhage also predicts brain injury in shaken baby syndrome [41]; it has been reported that retinal hemorrhage can rarely occur without intracranial hemorrhage or cerebral edema [49,50]. A systematic review of the clinical and radiographic characteristics associated with abusive and nonabusive head trauma also revealed that retinal hemorrhage is significantly associated with abusive head trauma [36]. As such, it is important to include terms relevant to retinal hemorrhage in the search strategy.

A range of definitions for identifying TBI in children and youth using administrative databases will be proposed, depending on the purpose of surveillance activity. For instance, a more conservative definition with high specificity may be preferred in monitoring healthcare utilization for a more moderate to severely injured population. However, a broader definition may be more suitable for prevention efforts, where information on ‘near misses’ through the inclusion of ‘head’ versus brain codes may be informative. The strength of this protocol is that it will utilize a more comprehensive and systematic search strategy, which builds upon previous work [32] that includes grey literature, and will not be limited to articles published in English only.

It is acknowledged that a systematic review of the literature is not sufficient to identify the definitive ICD-10 codes to define TBI in this population. Future research should include systematic reviews on the association of ICD-10 codes S00 to S09 with brain injury, in order to assess the relationship between these conditions and TBI more accurately. This may also assist in determining the most appropriate definition of TBI in children and youth for research using healthcare administrative data. More importantly, studies assessing the validity and accuracy of case ascertainment in administrative data for identifying TBI in children and youth should be conducted. This was also recommended in a recent review of the optimal ICD codes to study neurological conditions [20]. Data quality of the Discharge Abstract Database, which captures acute care admissions in Canada, has been assessed using chart re-abstraction and indicated good agreement for nonclinical variables, moderate to substantial agreement for the most responsible diagnoses (the diagnosis most responsible for the acute care length of stay), and good sensitivity and specificity of S02 and S06 codes [51]. However, this information is not available for the other ICD-10 codes that may be associated with TBI. It is critical that the full range of codes that could potentially serve to improve data quality and for the planning of both prevention and treatment programs are validated. Moreover, it is important to have accurate numbers for surveillance activity, as underestimates have implications for planning of healthcare services for this population and influence allocation of resources. Therefore, continuous monitoring of coding practices is crucial and will facilitate improved definition of TBI in children and youth in healthcare administrative data.

## Abbreviations

CDC: Centers for disease prevention and control; ED: Emergency department; ICD: International classification of diseases; ICD-9: International classification of Diseases version 9; ICD-10: International classification of diseases version 10; TBI: Traumatic brain injury.

## Competing interests

The authors declare that they have no competing interests.

## Authors’ contributions

VC and AC conceptualized the study. VC formulated the methods and design and drafted the paper, and VC and PT conducted the literature review. VC, AC, and PT had significant input in the editing process of the paper and revised it critically for important intellectual content. VC, AC, and PT read and approved the final manuscript.

## Supplementary Material

Additional file 1 Search strategy. Click here for file

Additional file 2 Table templates. Click here for file
