# Supplementary material for: Defining traumatic brain injury in children and youth using International Classification of Diseases version 10 codes: a systematic review protocol
Source: Syst Rev. 2013 Nov 13;2:102. doi: 10.1186/2046-4053-2-102 (PMC3833640; doi:10.1186/2046-4053-2-102)
Supplement: Additional file 1 — Search strategy. [file 2046-4053-2-102-S1.docx]

Additional files A

Additional files B: Eligibility criteria for screening studies

1. Is this study a randomized controlled trial?

YES _____

NO _____

UNCLEAR _____

2. Is the follow-up period of this study longer than 8 weeks?

YES _____

NO _____

UNCLEAR _____

3. Is the age of T2D patient in the RCT greater than18?

YES _____

NO _____

UNCLEAR _____

4. Is the intervention of this study dapagliflozin combined with conventional anti-diabetic drugs?

YES _____

NO _____

UNCLEAR _____

5. Is the control of this study placebo with conventional anti-diabetic drugs?

YES _____

NO _____

UNCLEAR _____

6. Does this study include any of these three outcomes: HbA1c, FPG, and body weight?

YES _____

NO _____

UNCLEAR _____

If you answer NO to any of these questions, the study will be excluded

**Additional files C**

**Table 1.** Basic characteristics of randomized controlled trials included in the systematic review.

| Author (year) | Intervention | | Participants baseline characteristic | | | | | Follow-up period (max) | Conclusion |
| --- | --- | --- | --- | --- | --- | --- | --- | --- | --- |
|  | Control | experiment | *n* | Age | HbA1c (%) | BMI or weight (kg/m^2^ or kg) | FPG (mmol/L or mg/dl) |  |  |
| Study 1 |  |  |  |  |  |  |  |  |  |
| Study 2 |  |  |  |  |  |  |  |  |  |
| Study 3 |  |  |  |  |  |  |  |  |  |
| Study 4 |  |  |  |  |  |  |  |  |  |
| …… |  |  |  |  |  |  |  |  |  |

**Additional files D**

**Table 2.** The Cochrane Collaboration’s tool for assessing risk of bias.

| Source of bias | Review authors’ judgment | RCT 1 | RCT 2 | RCT 3 | … |
| --- | --- | --- | --- | --- | --- |
| Random sequence generation | Selection bias due to inadequate generation of a randomized sequence | low/unclear/high | low/unclear/high | low/unclear/high |  |
| Allocation concealment | Selection bias due to inadequate concealment of allocations before assignment | low/unclear/high | low/unclear/high | low/unclear/high |  |
| Blinding of participants and personnel^a^ | Performance bias due to knowledge of the allocated interventions by participants and personnel during the study | low/unclear/high | low/unclear/high | low/unclear/high |  |
| Blinding of outcome assessment^a^ | Detection bias due to knowledge of the allocated interventions by outcome assessment | low/unclear/high | low/unclear/high | low/unclear/high |  |
| Incomplete outcome data^a^ | Attrition bias due to amount, nature, or handling of incomplete outcome data | low/unclear/high | low/unclear/high | low/unclear/high |  |
| Selective reporting | Reporting bias due to selective outcome reporting | low/unclear/high | low/unclear/high | low/unclear/high |  |

^a^Assessments should be made for each main outcome or class of outcomes.
