# Supplementary material for: Defining traumatic brain injury in children and youth using International Classification of Diseases version 10 codes: a systematic review protocol
Source: Syst Rev. 2013 Nov 13;2:102. doi: 10.1186/2046-4053-2-102 (PMC3833640; doi:10.1186/2046-4053-2-102)
Supplement: Additional file 2 — Table templates. [file 2046-4053-2-102-S2.pdf]

Chan V, Thurairajah T, Colantonio A.
Protocol

Additional file 2. Table templates

Table 1. Summary of Identified Peer-Reviewed Articles & Grey Literature Reports

Article (Author, Year)	Source of Data	Year of Studies	Location of Studies	Age of Population	Range of Incidence	ICD-10 Codes Validated?
mTBI						
TBI						
Severe TBI						
Head Injury						
Intentional Injury						

Table 2. Case Definition by Articles/Grey Literature Reports

ICD-10 Code	Article (Author, Year)																			
S00 Superficial Injury of Head																				
S00.0																				
S00.1																				
S00.2																				
S00.3																				
S00.4																				
S00.5																				
S00.6																				
S00.7																				
S00.8																				
S00.9																				
S01 Open Wound of Head																				
S01.0																				
S01.2																				
S01.2																				
S01.3																				
S01.4																				
S01.5																				
S01.7																				
S01.8																				
S01.9																				
S02 Fracture of Skull and Facial Bones																				
S02.0																				
S02.1																				
S02.2																				
S02.3																				
S02.4																				
S02.5																				
S02.6																				
S02.7																				
S02.8																				
S02.9																				
S03 Dislocation, Sprain, and Strain of Joints and Ligaments of Head																				
S03.0																				
S03.1																				
S03.2																				
S03.3																				
S03.4																				
S03.5																				
S04 Injury of Cranial Nerves																				
S04.0																				
S04.1																				
S04.2																				
S04.3																				
S04.4																				
S04.5																				

ICD-10 Code	Article (Author, Year)																			
S04.6																				
S04.7																				
S04.8																				
S04.9																				
S05 Injury of Eye and Orbit																				
S05.1																				
S05.2																				
S05.3																				
S05.4																				
S05.5																				
S05.6																				
S05.7																				
S05.8																				
S05.9																				
S06 Intracranial Injury																				
S06.0																				
S06.1																				
S06.2																				
S06.3																				
S06.4																				
S06.5																				
S06.6																				
S06.7																				
S06.8																				
S06.9																				
S07 Crushing Injury of Head																				
S07.0																				
S07.1																				
S07.8																				
S07.9																				
S08 Traumatic Amputation of Part of Head																				
S08.0																				
S08.1																				
S08.8																				
S08.9																				
S09 Other and Unspecified Injuries of Head																				
S09.0																				
S09.1																				
S09.2																				
S09.7																				
S09.8																				
S09.9																				
Injuries Involving Multiple Body Regions																				
T01.0																				
T02.0																				
T04.0																				
T06.0																				
T90 Sequelae of Injuries																				

ICD-10 Code	Article (Author, Year)																			
T90.0																				
T90.1																				
T90.2																				
T90.3																				
T90.4																				
T90.5																				
T90.8																				
T90.9																				
